# Optimizing Perioperative Management of Haemophilia B With rFIX‐FP: Pharmacokinetic Validation of the Hemoptidose Tool

**DOI:** 10.1111/hae.70326

**Published:** 2026-06-02

**Authors:** Xavier Delavenne, Célia Vollard, Nicolas Drillaud, Sabine Castet, Nicolas Luyck, Emilie Hénin, Julien Denis‐Le Seve, Fabienne Volot, Brigitte Tardy, Stéphanie Désage, Benoît Guillet

**Affiliations:** ^1^ Laboratoire De Pharmacologie et Toxicologie CHU de Saint‐Etienne Saint‐Priest‐en‐Jarez France; ^2^ INSERM UMR1059 SAINBIOSE, Equipe Dysfonction Vasculaire et de l'Hémostase Université Jean‐Monnet Saint‐Priest‐en‐Jarez France; ^3^ Irset (Institut De Recherche En Santé, environnement et travail) ‐ UMR_S 1085 Univ Rennes, CHU Rennes, Inserm, EHESP Rennes France; ^4^ CRMW, CRC‐MHC (Centre de Référence maladie de Willebrand, Centre de Ressource et de Compétence des Maladies Hémorragiques Constitutionnelles) Hôtel‐Dieu University Hospital Nantes France; ^5^ Haemophilia Treatment Center University Hospital Bordeaux France; ^6^ Calvagone Chazay d'Azergues France; ^7^ Haemophilia Treatment Center, Hospital Angers France; ^8^ Haemophilia Treatment Center University Hospital Dijon France; ^9^ CRC hémophilie et maladies hémorragiques, CIC 1408 CHU Saint Etienne Saint‐Étienne France; ^10^ CRH, CRC‐MHC (Centre de Référence de l'Hémophilie, Centre de Ressource et de Compétence des Maladies Hémorragiques Constitutionnelles) Hospices civils de Lyon Bron France; ^11^ CRH, CRC‐MHC (Centre de Référence de l'Hémophilie, Centre de Ressource et de Compétence des Maladies Hémorragiques Constitutionnelles) University Hospital Rennes France

**Keywords:** albutrepenonacog alfa, Bayesian estimation, haemophilia B, perioperative management, pharmacokinetics, rFIX‐FP

## Abstract

**Background:**

Surgical management of haemophilia B requires precise factor IX replacement to ensure adequate haemostasis while optimizing factor consumption. Extended half‐life rFIX‐FP simplifies perioperative management but exhibits substantial pharmacokinetic variability. PK‐guided dosing is recommended by WFH guidelines, yet validated tools for perioperative rFIX‐FP individualization are lacking.

**Objectives:**

To validate a population PK model under perioperative conditions and evaluate Hemoptidose, a model‐informed precision dosing (MIPD) platform, for individualizing rFIX‐FP therapy during surgery.

**Methods:**

We retrospectively analyzed 24 surgeries in haemophilia B patients treated with rFIX‐FP. Predictive performance was assessed at population level and after Bayesian individualization with varying numbers of FIX samples. Actual perioperative consumption was retrospectively compared with Hemoptidose‐recommended doses.

**Results:**

The population PK model demonstrated robust perioperative performance. Population predictions yielded a mean absolute error (MAE) of 20.6%; after Bayesian updating, accuracy improved with MAE of 10.0%. Prediction error decreased from 20.6% without monitoring to 14% with three samples. Retrospective analysis indicated a potential 49% reduction in factor consumption with model‐guided dosing compared to empirical regimens (p < 0.001).

**Conclusions:**

The population PK model retains valid predictive performance in surgical settings. Two to three FIX measurements enable reliable individualization. The substantial discrepancy between empirical and model‐guided dosing highlights major optimization potential. Hemoptidose provides accessible MIPD implementation for perioperative rFIX‐FP management.

## 1 Introduction

Surgical care in haemophilia B requires a careful balance: inadequate replacement exposes patients to perioperative bleeding, while excessive dosing increases costs and, rarely, may predispose to thrombotic complications [1, 2]. Individualized dosing may also facilitate earlier transition to home‐based self‐treatment, potentially reducing hospitalization duration. Perioperative management must achieve defined time‐varying FIX activity targets, typically aiming for peak pre‐incision levels in the 80–100 IU/dL range for major surgery (less for minor procedures), followed by step‐down maintenance (e.g., >50 IU/dL for 7–14 days after major surgery) [1]. Achieving these dynamic targets is complicated by substantial inter‐ and intra‐patient pharmacokinetic (PK) variability and by practical constraints on monitoring in the perioperative environment. Acute surgical physiology (inflammation, fluid shifts, capillary leak, blood loss, transfusion) can transiently modify both clearance and volume of distribution [3]. These factors make simple weight‐based nomograms imprecise for many individuals. Clinical risk stratification also varies by procedure. Orthopedic and major abdominal/thoracic surgeries demand sustained coverage through tissue healing, whereas dental and minor cutaneous procedures may require only brief coverage [1].

Extended half‐life recombinant FIX‐albumin fusion protein (rFIX‐FP, albutrepenonacog alfa) offers advantages in surgical management of haemophilia B [4]. Compared with standard FIX products, rFIX‐FP maintains higher and more sustained FIX activity with far fewer infusions, which is crucial in the perioperative setting where stable haemostasis is required. rFIX‐FP exhibits a markedly prolonged terminal half‐life of approximately 90–105 h in adults and an incremental recovery of approximately 1.3 IU/dL per IU/kg, representing a 25%–45% improvement over standard half‐life FIX products [5, 6]. These properties result in higher and more sustained plasma FIX activity with fewer infusions. In the PROLONG‐9FP surgical substudy, which included 30 procedures in 21 patients, a single preoperative bolus of rFIX‐FP was sufficient in nearly all cases, with excellent or good haemostatic efficacy reported in >95% of surgeries [3]. Postoperative management was simplified, with a median of only five additional infusions over 14 days after major surgery and low overall factor consumption (median ∼222 IU/kg) [3]. These benefits have been confirmed in real‐world practice: Spanish and Italian cohorts showed that switching to rFIX‐FP prophylaxis reduced infusion frequency by 50–80%, decreased annual factor consumption by ∼60%, and maintained higher trough FIX levels (>5%–7%), while preserving excellent bleed protection [7, 8].

However, as with other recombinant FIX products, its PK variability remains substantial. In their population PK analysis of rIX‐FP, Zhang and colleagues identified body weight as a significant covariate for clearance and volume of distribution but also noted persistent inter‐individual variability beyond body weight effects [6]. Additional determinants such as baseline endogenous FIX activity and clinical context (e.g., perioperative fluid shifts, blood loss, transfusion, or systemic inflammation) can further modify exposure. This supports the need for individualized dosing strategies rather than reliance on fixed weight‐based regimens [3, 4]. Additionally, FIX activity monitoring is complicated by reagent‐dependent variability of one‐stage clotting assays with rFIX‐FP, and the fact that chromogenic assays overestimate FIX activity with rFIX‐FP, making silica‐based one‐stage clotting assays the preferred monitoring method for this product [9, 10].

Although thrombotic events with FIX replacement are uncommon, modern EHL concentrates capable of sustaining high levels for days introduce a theoretical risk of prolonged supratherapeutic exposure, particularly if early perioperative dose stacking is not adapted to the patient's PK [11].

Given these challenges, PK‐informed dosing is increasingly recommended to bridge the gap between guideline targets and patient‐specific needs [1]. For rFIX‐FP, individualized PK allows clinicians to (i) compute a pre‐incision loading dose that reliably attains the desired peak, (ii) schedule maintenance doses that maintain time‐in‐target while avoiding unnecessary overexposure, and (iii) adapt rapidly when measured FIX results become available. Bayesian estimation frameworks are particularly useful in the perioperative setting: they leverage a validated population model and patient covariates to generate accurate a priori predictions in the absence of levels, then assimilate sparse a posteriori measurements to refine individual clearance and recovery in real time [12].

The aim of this study was to validate a population PK model under perioperative conditions and to evaluate Hemoptidose, an open‐source model‐informed precision dosing (MIPD) platform, for individualizing rFIX‐FP therapy during surgery.

## 2 Materials and Methods

### 2.1 Study Design and Population

This was a retrospective, observational, multicenter study conducted within the BERHLINGO haemostasis research network in France. We included patients with haemophilia B (any severity) who underwent elective or urgent surgery managed with rFIX‐FP (albutrepenonacog alfa, Idelvion) and for whom at least one perioperative FIX activity measurement was available. To increase sample size given the rarity of haemophilia B surgical procedures, data were also pooled from two descriptive cohort studies of rFIX‐FP (IDEFIX and ORPHEE). Patients were excluded if they had active inhibitors against FIX or if dosing records were incomplete. Although no single standardized perioperative dosing protocol was imposed across participating centres, all followed WFH guidelines for perioperative haemophilia management [1], including systematic FIX activity monitoring to guide dose adjustments throughout the post‐surgical period. The study protocol was approved by the Ethics Committee of the University Hospital of Rennes (approval No. 25.88), and patients were informed with a written information notice. All procedures were conducted in accordance with the Declaration of Helsinki and applicable French regulatory requirements for retrospective clinical research.

### 2.2 Data Collection and FIX Activity Measurements

Clinical data were extracted from medical records and included patient demographics (age, sex, body weight), haemophilia severity (baseline FIX activity), surgical procedure characteristics (type, bleeding risk classification), rFIX‐FP dosing history (dose and timing of each infusion), perioperative FIX activity measurements (timing, assay method), and clinical outcomes (bleeding complications, thrombotic events).

FIX activity was measured using one‐stage clotting assays with silica‐based activated partial thromboplastin time (aPTT) reagents: Pathromtin SL (Siemens), SynthASil (Werfen), or aPTT‐SP (Table 1). Silica‐based one‐stage assays are recommended for rFIX‐FP monitoring, as chromogenic assays overestimate FIX activity with this product [9, 10]. Sample timing relative to rFIX‐FP infusions was recorded to allow accurate PK modeling.

**TABLE 1:** Population description.

	Mean ± SD, [min—max] or *n*, (%)
*N* surgery	24
Severity of haemophilia	
Mild	11 (45.8%)
Moderate	9 (37.5%)
Severe	4 (16.7%)
Age (years)	43.1 ± 22.5 [6–82]
Body weight (Kg)	76.0 ± 30.5 [19–180]
Height (cm)	171.9 ± 17.4 [116–190]
Number of samples per patient	4.5 ± 2.9 [1–14]
Number of injections	3.1 ± 2.1 [1–10]
Reagents (*n* samples)	
APTT	1 (3.0%)
Pathromtin SL	7 (21.2%)
SynthAsil	25 (75.8%)

### 2.3 Population PK Model

The Hemoptidose tool is based on the population pharmacokinetic (PopPK) model of rFIX‐FP developed by Zhang et al. [6]. This model was derived from four pivotal clinical trials (PROLONG‐9FP program) including 132 pediatric, adolescent, and adult patients with severe or moderately severe haemophilia B treated in prophylactic or on‐demand settings. It has a two‐compartment structure with first‐order elimination, interindividual variability on clearance (CL) and central volume of distribution (V1), and a combined proportional and additive residual error model. Body weight was implemented as an allometric covariate on CL and distribution volumes. No surgical patients were included in the original model development dataset.

### 2.4 Hemoptidose Tool

Hemoptidose is an open‐source, Shiny‐based web application designed to support model‐informed precision dosing (MIPD) of rFIX‐FP in haemophilia B surgical settings. The platform provides: (i) a priori simulations, allowing clinicians to predict FIX activity profiles from patient covariates (body weight) and a proposed dosing regimen; (ii) a posteriori Bayesian estimation of individual PK parameters, incorporating observed perioperative FIX measurements to refine predictions; and (iii) individualized dose optimization to achieve predefined FIX activity targets.

The application is implemented in R (version 4.x) using the Campsis framework for PK/PD simulations and relies on external engines (rxode2 and mrgsolve) to solve the underlying differential equations [13]. Individual Bayesian PK parameters are estimated using the maximum a posteriori probability (MAP) method [14]. The MAP estimate is defined as:

$$\;{\hat{\theta }}_{MAP}=\arg\max_{\theta }p\left(\theta |y\right)\propto arg\mathrm{ma}x_{\theta }\left[p\left(y|\theta \right)\cdot p\left(\theta \right)\right]$$

where y denotes the observed FIX activities, θ the vector of individual PK parameters, p(y|θ) the likelihood function, and p(θ) the population prior from the Zhang PopPK model. The estimator was implemented using the campsismap R package (version 0.6.1) [15].

**FIGURE 1:**
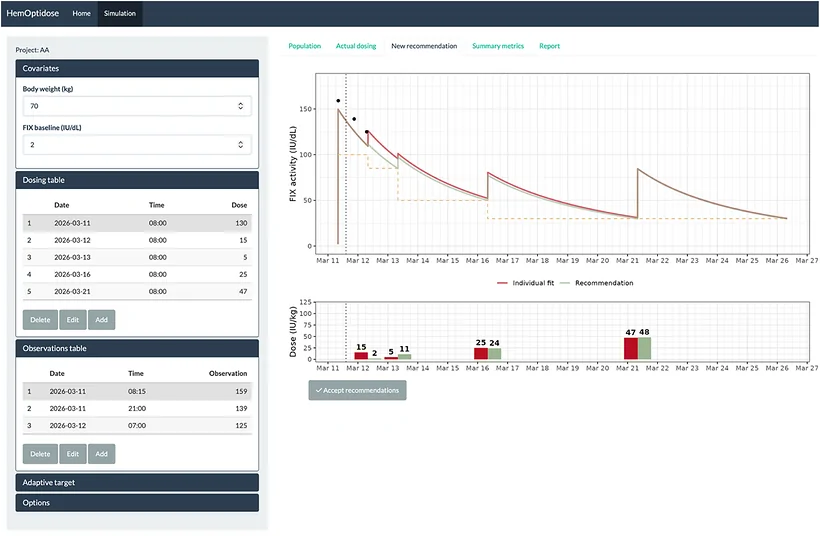
Hemoptidose user interface showing the dose recommendation module. Left panel: Patient covariates (body weight, baseline FIX activity), dosing table listing administered rFIX‐FP infusions (date, time, dose in IU/kg), and observations table displaying measured FIX activity levels with sampling times. Right panel: The 'New recommendation' tab shows the predicted FIX activity profile over time. The upper graph displays the individual Bayesian fit (red line) based on observed measurements (black dots) and the predicted profile with recommended doses (green line), with step‐down target ranges indicated in the background. The lower graph shows the corresponding dosing regimen, comparing administered doses (red bars) with tool‐recommended doses (green bars). The 'Accept recommendations' button allows integration of optimized doses into the treatment plan.

For data protection, all calculations are performed locally on the user's device; no information is transmitted to external servers. Projects can be exported, archived, and re‐imported as treatment evolves. The user interface is illustrated in Figure 1.

### 2.5 Validation of Model Predictive Performance

Because perioperative physiology may differ from prophylaxis or on‐demand conditions, the applicability of the Zhang PopPK model was evaluated in surgical patients. Observed perioperative FIX activity levels were compared with model‐predicted concentrations using both a priori (population‐based, without individual FIX data) and a posteriori (Bayesian individualized) approaches. Predictive performance was assessed using (i) goodness‐of‐fit plots comparing observed FIX levels to population and individual predictions, (ii) Bland–Altman plots to evaluate agreement between platforms, and (iii) quantitative metrics including bias (mean prediction error), mean absolute error (MAE), and root mean square error (RMSE). To confirm computational accuracy, Hemoptidose parameter estimates and predictions were compared against Monolix (version 2021R1, Lixoft), the reference software for population PK analysis [16].

### 2.6 Impact of Sampling Strategy

We evaluated the impact of the number of FIX activity measurements on prediction accuracy. Predictive performance was assessed under a priori conditions (using only patient covariates without FIX monitoring) and then under a posteriori conditions, progressively incorporating one to seven perioperative FIX measurements into the Bayesian estimation. This analysis quantified how additional sampling improved prediction reliability.

### 2.7 Comparison of Observed versus Model‐recommended Factor Consumption

We compared administered rFIX‐FP doses with doses that Hemoptidose would have recommended to achieve perioperative FIX targets. For each surgical procedure, cumulative observed factor consumption (IU/kg) was calculated and compared with Hemoptidose‐recommended regimens targeting the same FIX activity goals.

### 2.8 Statistical Analysis

Continuous variables are presented as median (range) or mean ± standard deviation, as appropriate. Categorical variables are presented as counts and percentages. Agreement between Hemoptidose and Monolix was assessed using Bland–Altman analysis, with limits of agreement defined as mean bias ± 1.96 SD. Observed and recommended factor consumption were compared using the Wilcoxon signed‐rank test. A two‐sided *p*‐value <0.05 was considered statistically significant. Statistical analyses were performed using R (version 4.x).

## 3 Results

### 3.1 Study Population

Twenty‐four surgical procedures in 22 patients with haemophilia B were included in the analysis (Table 1). Surgical specialties included orthopedic (*n* = 8), digestive (*n* = 9), Dental/maxillofacial (*n* = 5), vascular (*n* = 1), and urological (*n* = 1) procedures. Among orthopaedic procedures, 5 were total knee replacements. Digestive procedures included sleeve gastrectomy, gastric bypass, haemorrhoidectomy, hernia repair, and emergency abdominal surgeries. Dental/maxillofacial procedures included wisdom teeth extractions (*n* = 2), mandibular advancement, and genioplasty. Among the 24 surgeries, 4 (16.7%) were performed in patients with severe haemophilia B (baseline FIX < 1%), 9 (37.5%) with moderate disease (FIX 1%–5%), and 11 (45.8%) with mild disease (FIX > 5%). According to WFH classification [1], 23 procedures (95.8%) were classified as major/high‐risk surgeries, with only one minor procedure. Mean patient age was 43.1 ± 22.5 years (range: 6–82 years) and mean body weight was 76.0 ± 30.5 kg (range: 19–180 kg). During the perioperative period, patients received a median of 3 rFIX‐FP infusions (range: 1–10). A total of 113 FIX activity samples were collected, with a mean of 4.7 samples per surgical procedure (range: 1–14).

### 3.2 Predictive Performance of the Zhang PopPK Model

**FIGURE 2:**
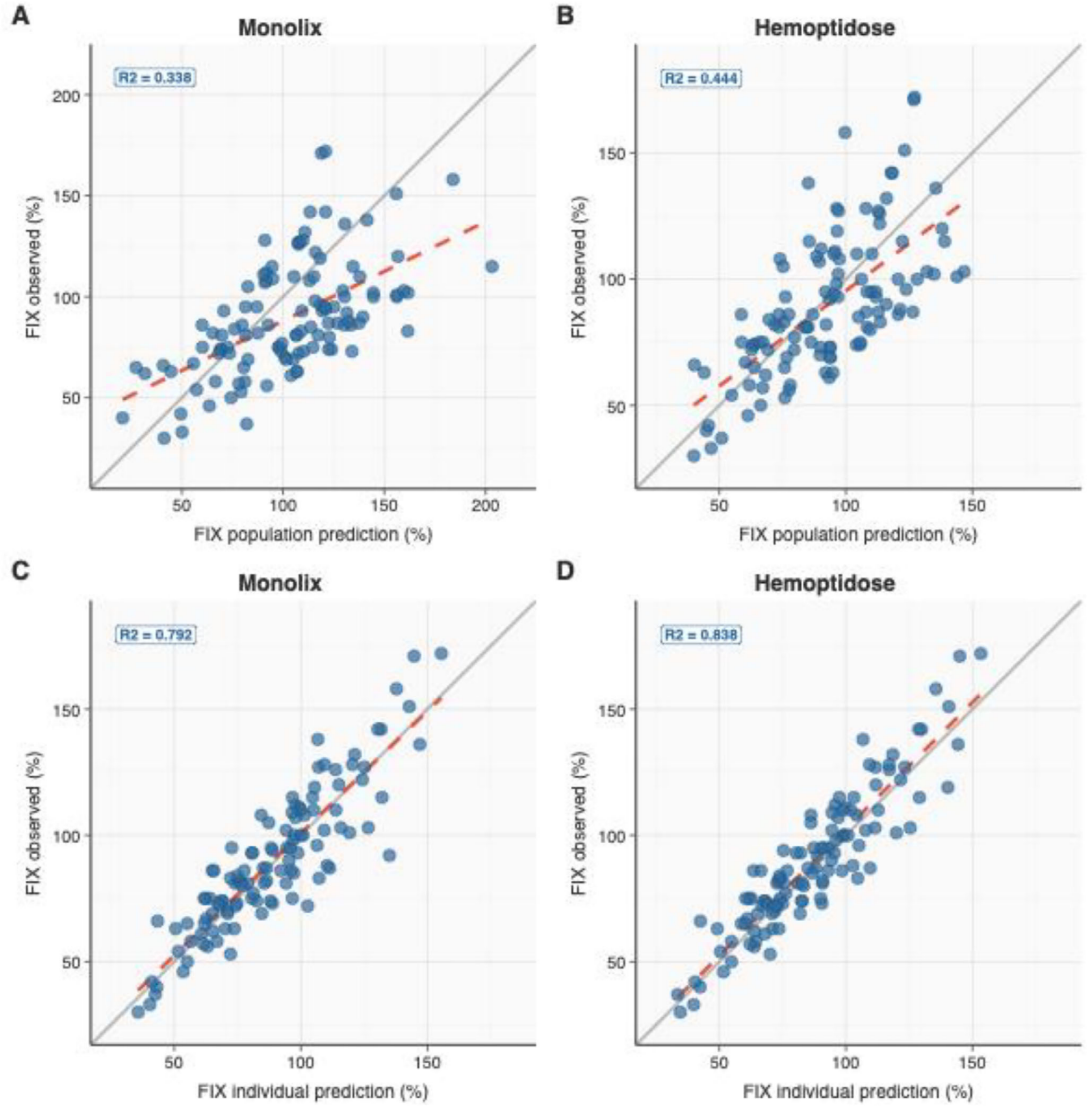
Goodness‐of‐fit plots comparing observed FIX activity levels (%) with population predictions (A, B) and individual predictions (C, D) from Monolix (A, C) and Hemoptidose (B, D). The grey solid line represents the line of identity, and the red dashed line represents the linear regression. R^2^ values are displayed for each panel.

The perioperative applicability of the Zhang rFIX‐FP PopPK model was assessed using both Monolix and Hemoptidose. Goodness‐of‐fit plots are presented in Figure 2.

Population‐level predictions. Population‐level predictions (without individual FIX data) showed substantial dispersion compared with observed FIX concentrations (Figure 2). The regression showed a trend toward underestimation at low concentrations and overestimation at higher concentrations, reflecting the intrinsic inter‐individual variability expected from a priori predictions based solely on body weight. At the population level, MAE was 20.6%, RMSE was 25.0%, and the coefficient of determination was *R* ^2^ = 0.38, consistent with published PopPK models in haemophilia [12, 17]. These a priori predictions serve as a starting point for initial dose planning, prior to any FIX monitoring.

Individual‐level predictions after Bayesian updating. After incorporation of observed FIX measurements through Bayesian estimation, goodness‐of‐fit improved markedly (Figure 2). For Monolix, R^2^ increased from 0.38 at the population level to 0.79 at the individual level, with MAE decreasing to 10.0% and RMSE to 12.8%. Similar performance was observed with Hemoptidose (MAE = 9.2%, RMSE = 12.8%), with 77% of predictions falling within ±15% of observed FIX levels.

### 3.3 Agreement Between Hemoptidose and Monolix

**FIGURE 3:**
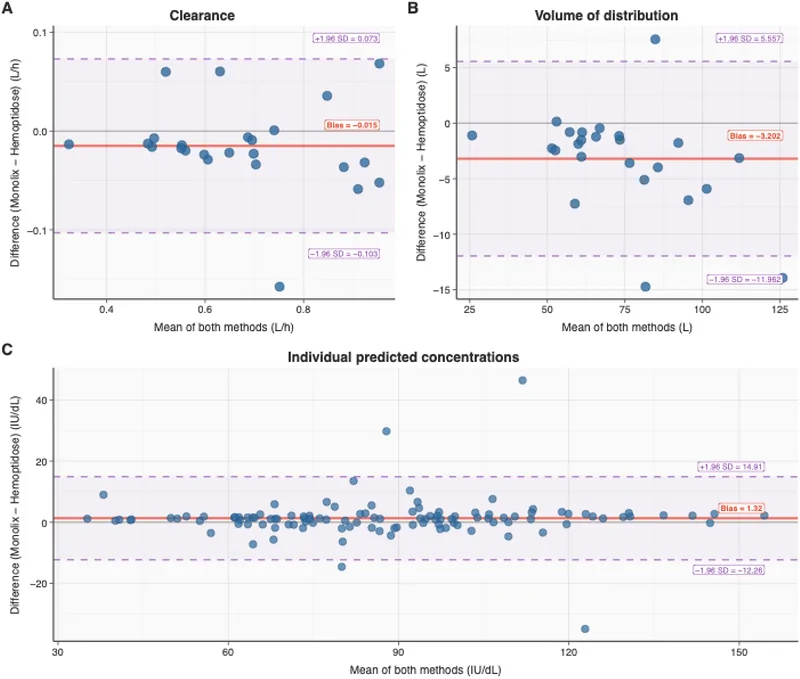
Bland–Altman plots assessing agreement between Monolix and Hemoptidose for individual estimates of clearance (A), volume of distribution (B), and predicted FIX activity concentrations (C). The red solid line indicates the mean bias, and the purple dashed lines indicate the 95% limits of agreement (±1.96 SD). The shaded area represents the 95% agreement interval.

Bland–Altman analysis showed close agreement between Hemoptidose and Monolix for individual PK parameter estimates (Figure 3). For clearance (CL), mean bias was +0.01 L/h with limits of agreement of −0.07 to +0.10 L/h (MAE 0.03 L/h, RMSE 0.05 L/h). For central volume of distribution (V1), Hemoptidose showed a slight systematic overestimation relative to Monolix (+3.2 L; limits of agreement: −5.6 to +11.9 L; MAE 3.8 L, RMSE 5.4 L).

### 3.4 Impact of Sampling Strategy on Predictive Accuracy

**FIGURE 4:**
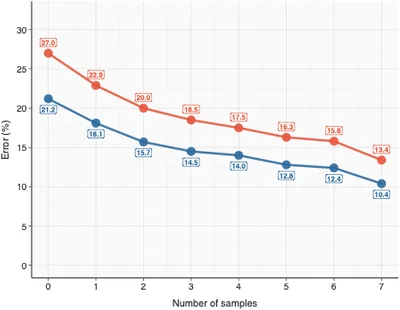
Impact of the number of available FIX activity samples on prediction accuracy, expressed as MAE (blue line) and RMSE (red line). Values (%) are displayed for each data point.

The predictive performance of Hemoptidose was evaluated according to the number of perioperative FIX measurements incorporated into the Bayesian estimation (Figure 4). Without FIX monitoring (a priori predictions based solely on body weight), MAE was 20.6% and RMSE was 25%. Prediction accuracy improved with each additional sample: after two measurements, MAE decreased to 16% (RMSE 19%); after three samples, MAE reached 14% (RMSE 17%) and remained stable thereafter.

### 3.5 Comparison of Observed versus Model‐recommended Factor Consumption

**FIGURE 5:**
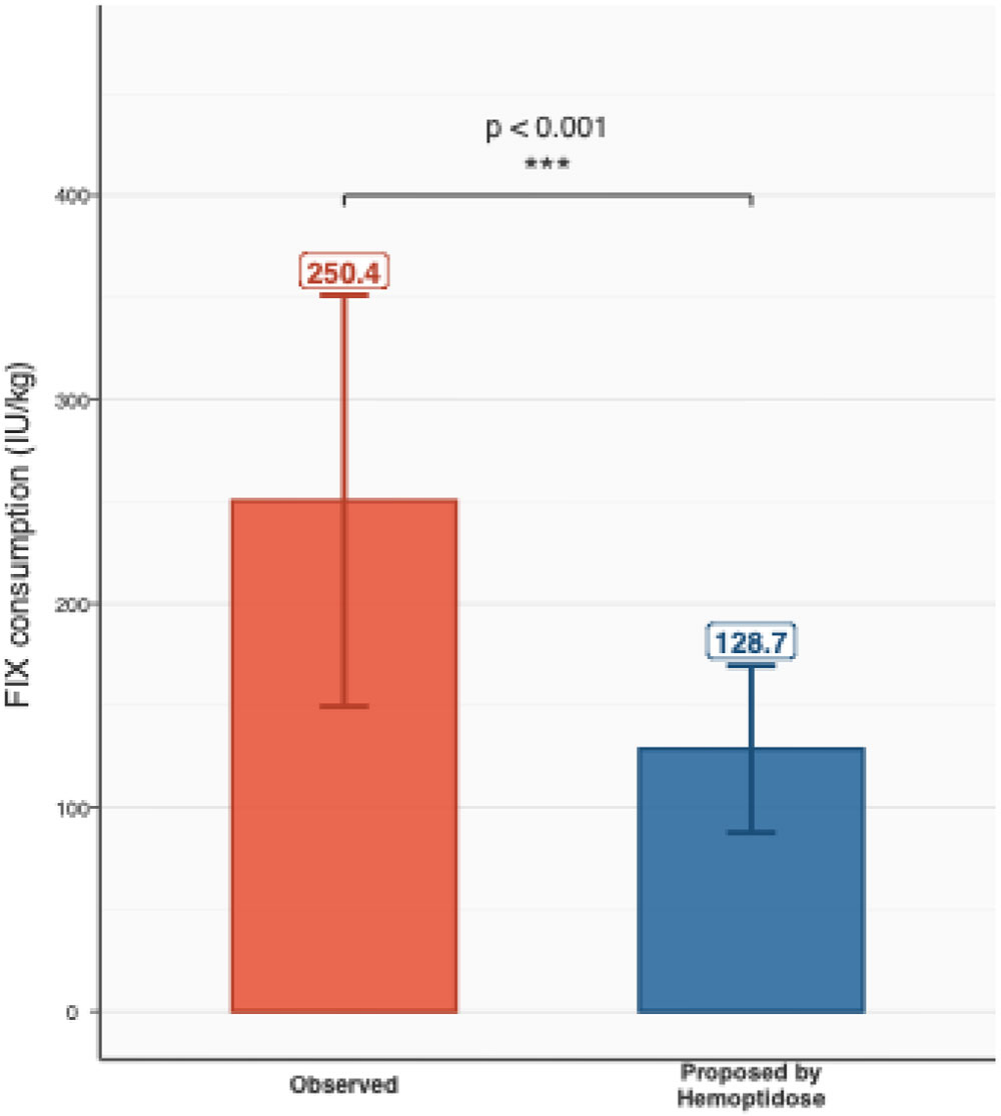
Comparison of total perioperative FIX concentrate consumption (IU/kg) between observed clinical practice and Hemoptidose‐recommended dosing regimens. Error bars represent ±1 SD. *** p < 0.001 (paired Wilcoxon signed‐rank test).

Observed and Hemoptidose‐recommended rFIX‐FP consumption were compared for each surgical procedure (Figure 5). Observed perioperative consumption averaged 250.4 ± 100.7 IU/kg per procedure. Hemoptidose‐recommended regimens targeting the same FIX activity thresholds would have required 128.7 ± 40.9 IU/kg, a 49% reduction (p < 0.001, Wilcoxon signed‐rank test).

## 4 Discussion

This study provides the first clinical validation of Hemoptidose, a MIPD tool designed for perioperative management of rFIX‐FP in haemophilia B. Using retrospective data from 24 surgical procedures, we showed that the Zhang population PK model [6] retains robust predictive performance under surgical conditions and that Hemoptidose achieves Bayesian estimation accuracy equivalent to Monolix while providing a user‐friendly, secure interface suitable for routine clinical use.

The primary objective of perioperative dosing is to ensure adequate haemostatic coverage and patient safety. A clinically meaningful finding concerns factor consumption optimization. Retrospective comparison of administered doses with Hemoptidose recommendations revealed that observed perioperative FIX consumption averaged 250.4 IU/kg, whereas the tool would have recommended 128.7 IU/kg (p < 0.001), a potential 49% reduction. Perioperative overdosing is a well‐documented phenomenon. In the OPTI‐CLOT programme, Hazendonk et al. showed that 33%–75% of achieved FVIII levels exceeded target ranges in severe haemophilia A surgery, with a potential 44% reduction if levels had been maintained within targets [18]. Schütte et al. confirmed similar findings in mild haemophilia A, with 65% of perioperative FVIII measurements above target [19]. A perioperative PopPK model was subsequently developed to address this overexposure through Bayesian adaptive dosing [20]. In the prophylactic setting, Carlsson and colleagues reported 30%–43% reductions in factor consumption through PK‐individualized dosing regimens [21, 22]. While this analysis is retrospective and cannot account for all clinical considerations influencing prescribing decisions, it shows the magnitude of potential optimization achievable through model‐informed dosing. For high‐cost extended half‐life products such as rFIX‐FP, such optimization could translate into meaningful savings without compromising haemostatic efficacy [3, 4]. Beyond cost considerations, avoiding unnecessary overexposure may also have safety implications. Extended half‐life products can sustain elevated FIX levels for several days, which may increase the risk of prolonged supratherapeutic exposure if doses are not individualized [11, 23]. By predicting individual FIX trajectories, Hemoptidose allows clinicians to identify patients at risk of overexposure and adjust maintenance dosing accordingly. In our cohort, no thrombotic or bleeding complications were reported, although this study was not designed to capture safety outcomes systematically.

We also evaluated the number of FIX measurements required for reliable Bayesian individualization. Our analysis shows that prediction accuracy improves with each additional sample but with diminishing returns beyond two to three measurements. Without FIX monitoring, MAE was 20.6%, decreasing to 16% with two samples and reaching a plateau around 14% thereafter. These results are consistent with recent evaluations of PK‐guided prophylactic dosing in haemophilia, where Bayesian individualization with limited sampling achieved clinically adequate predictive performance [17]. These findings support a sparse sampling strategy, such as a pre‐incision level followed by one or two postoperative measurements, which aligns with current WFH guidelines recommending PK‐guided dosing [1] while remaining feasible in routine perioperative care [24, 25].

Hemoptidose offers several features relevant to surgical haemophilia management. The platform allows real‐time Bayesian updating as FIX results become available, allowing dose adjustments throughout the perioperative period. All calculations are performed locally without data transmission to external servers, addressing institutional data governance requirements. Projects can be exported, archived, and re‐imported as treatment evolves, facilitating continuity across the surgical episode. The tool also generates structured clinical reports documenting dosing rationale, which supports multidisciplinary communication between hematologists, anesthesiologists, and surgeons. Hemoptidose is freely available and based on open‐source components (R, Campsis, campsismap) [13, 15], promoting accessibility and reproducibility.

This study has limitations inherent to its retrospective design and modest sample size, reflecting the rarity of haemophilia B surgical procedures. The study population was heterogeneous in terms of age (6–82 years), body weight (19–180 kg), surgical type, and treatment intensity. While this may limit subgroup‐specific conclusions, it reflects the clinical reality of haemophilia B surgery and demonstrates the model's robustness across diverse real‐world conditions. The Zhang model was developed from prophylaxis and on‐demand data and does not include surgery‐specific covariates such as blood loss or inflammation, which may transiently alter FIX PKs [3, 6]. FIX assay variability, particularly with one‐stage clotting methods, introduces measurement uncertainty that propagates into Bayesian estimates [9, 10]. Clinical endpoints such as bleeding events or length of stay were not assessed, as the primary aim was PK validation. Any future adoption of PK‐guided perioperative dosing should be validated prospectively with clinical endpoints, including bleeding events and target attainment, before changes to surgical protocols are considered.

Future prospective studies should evaluate whether systematic hemoptidose use improves target attainment, reduces factor consumption, and affects clinical outcomes compared to empirical dosing. Integration with existing population PK platforms such as WAPPS‐Hemo [26] and model refinement incorporating perioperative covariates and extension to other extended half‐life FIX products would broaden clinical utility [27].

In conclusion, hemoptidose allows individualized, PK‐guided perioperative dosing of rFIX‐FP with accuracy equivalent to reference pharmacometric software. The gap between observed and recommended factor consumption shows the optimization potential in current surgical practice. By combining scientific rigor with practical accessibility, hemoptidose is a clinically relevant tool for precision dosing in haemophilia B surgery.

## Author Contributions

All authors contributed to the design of the study, the acquisition of data, and the interpretation of data. X.D. and B.G. contributed to the conception of the study and the analysis of data. All authors commented on all intermediary versions of the manuscript. All authors read and approved the final version of the manuscript to be submitted.

## Funding

The authors have nothing to report.

## Ethic Statement

This Research Was Conducted in Accordance With the Principles of the Declaration of Helsinki. It Has Been Approved by the Institutional Review Board and Ethics Committee

## Conflicts of Interest

Xavier Delavenne has received honoraria for participation in symposia from CSL Behring, Shire, Octapharma, LFB Biopharmaceuticals, and Sobi Brigitte Tardy has received funds for research from Baxalta, CSL Behring, Novo Nordisk, Octapharma, Pfizer, Roche, and Sobi and honoraria from Pfizer, Novo Nordisk, and Sobi. Sabine‐Marie Castet has been a consultant for Shire/Takeda, CSL Behring, LFB Biopharmaceuticals, Novo Nordisk, Roche‐Chugai, Sobi, and Pfizer. Fabienne Volot has been a consultant for Sobi, Roche, Pfizer, LFB Biopharmaceuticals, and Takeda. Benoît Guillet has been a consultant for Baxter/Baxalta/Shire/Takeda, CSL Behring, LFB Biopharmaceuticals, Novo Nordisk, Octapharma, Roche‐Chugai, and Sobi. R.d'O. reports honoraria for lectures or advisory boards from BioMarin, CSL Behring, LFB, NovoNordisk, Octapharma, Pfizer, Roche/Chugai, Sobi/Sanofi, and Takeda.

## Data Availability

Data are available from the corresponding author upon reasonable request.
